# Mechanisms regulating neuronal excitability and seizure development following mTOR pathway hyperactivation

**DOI:** 10.3389/fnmol.2014.00018

**Published:** 2014-03-14

**Authors:** Candi L. LaSarge, Steve C. Danzer

**Affiliations:** ^1^Department of Anesthesia, Cincinnati Children’s Hospital Medical CenterCincinnati, OH, USA; ^2^Department of Anesthesia, University of CincinnatiCincinnati, OH, USA; ^3^Department of Pediatrics, University of CincinnatiCincinnati, OH, USA

**Keywords:** granule cells, epilepsy, mTOR, neurogenesis, PTEN, TSC, hippocampus, autism

## Abstract

The phosphatidylinositol-3-kinase/phosphatase and tensin homolog (PTEN)-mammalian target of rapamycin (mTOR) pathway regulates a variety of neuronal functions, including cell proliferation, survival, growth, and plasticity. Dysregulation of the pathway is implicated in the development of both genetic and acquired epilepsies. Indeed, several causal mutations have been identified in patients with epilepsy, the most prominent of these being mutations in PTEN and tuberous sclerosis complexes 1 and 2 (TSC1, TSC2). These genes act as negative regulators of mTOR signaling, and mutations lead to hyperactivation of the pathway. Animal models deleting PTEN, TSC1, and TSC2 consistently produce epilepsy phenotypes, demonstrating that increased mTOR signaling can provoke neuronal hyperexcitability. Given the broad range of changes induced by altered mTOR signaling, however, the mechanisms underlying seizure development in these animals remain uncertain. In transgenic mice, cell populations with hyperactive mTOR have many structural abnormalities that support recurrent circuit formation, including somatic and dendritic hypertrophy, aberrant basal dendrites, and enlargement of axon tracts. At the functional level, mTOR hyperactivation is commonly, but not always, associated with enhanced synaptic transmission and plasticity. Moreover, these populations of abnormal neurons can affect the larger network, inducing secondary changes that may explain paradoxical findings reported between cell and network functioning in different models or at different developmental time points. Here, we review the animal literature examining the link between mTOR hyperactivation and epileptogenesis, emphasizing the impact of enhanced mTOR signaling on neuronal form and function.

## INTRODUCTION

The mammalian target of rapamycin (mTOR) is a serine/threonine kinase involved in the highly conserved phosphatidylinositol-3-kinase (PI3K)-Akt signaling pathway. mTOR regulates neuronal proliferation, survival, growth, and functioning (Sarbassov et al., 2005). Since this pathway is critical to development, dysregulation at any stage can have deleterious consequences (Tokuda et al., 2011). Mutations or deletions of phosphatase and tensin homolog (PTEN) and the heterodimeric complex of tuberous sclerosis proteins 1 and 2 (TSC1/hamartin, TSC2/tuberin) can dramatically upregulate mTOR signaling and contribute to a class of human neurological diseases characterized as “TORopathies” (Crino, 2009; Wong and Crino, 2012). PTEN dysregulation has been linked to disorders that include Lhermitte–Duclos disease (Liaw et al., 1997), Cowden syndrome (Goffin et al., 2001), Proteus syndrome (Zhou et al., 2001), and Bannayan–Riley–Ruvalcaba syndrome (Arch et al., 1997; Eng, 2003). Additionally, PTEN mutations have been associated with a number of neurological conditions, such as epilepsy, macrocephaly, mental retardation, and autism spectrum disorders (Marsh et al., 1999; Goffin et al., 2001; Zhou et al., 2003; Butler et al., 2005; Herman et al., 2007; O’Roak et al., 2012; Epi4K Consortium and Epilepsy Phenome/Genome Project, 2013). Dysregulation of either TSC1 or TSC2 leads to TSC, a disorder characterized by the widespread development of non-malignant tumors in multiple organ systems, including the brain, eyes, heart, skin, and lungs (for review, see Franz et al., 2010). However, the central nervous system (CNS) manifestations of TSC are the most disabling and include seizures, mental retardation, and behavioral disorders like autism (Sparagana and Roach, 2000). Epilepsy is the most common neurological symptom of TSC, often developing within a year of birth and affecting 60–90% of individuals over their lifetime (Holmes and Stafstrom, 2007). Promisingly, the mTOR antagonist rapamycin and its analogs have already entered clinical trials for TSC, with initial studies showing encouraging results (Krueger and Franz, 2008; Muncy et al., 2009; Wong, 2010; Kotulska et al., 2013; Krueger et al., 2013). TORopathies may also include disorders with evidence of enhanced mTOR signaling, but lacking known mTOR pathway mutations. For example, enhanced mTOR signaling has been observed in focal cortical dysplasia (Chen et al., 2012; Lim and Crino, 2013; Wong, 2013b; Yasin et al., 2013) and mesial temporal sclerosis (Sha et al., 2012; Sosunov et al., 2012); although the role of enhanced signaling in these conditions remains uncertain.

**FIGURE 1:**
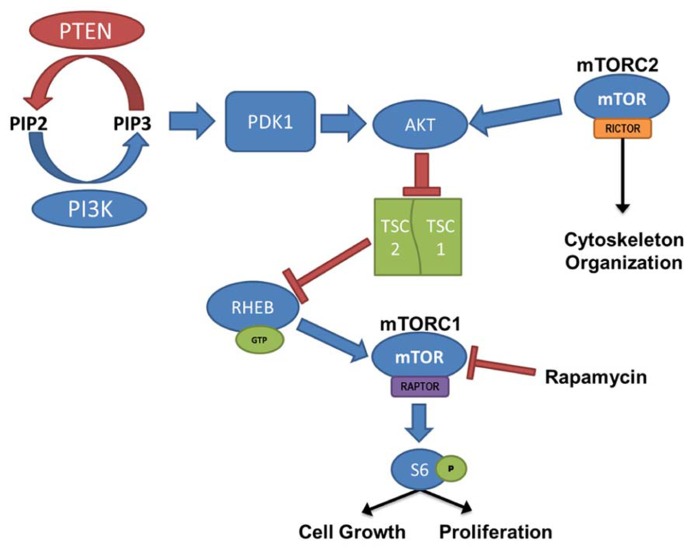
**PI3K/PTEN–mTOR pathway.** mTOR is activated by signaling through the PI3K–Akt pathway, regulating cell growth and proliferation. PTEN, TSC1, and TSC2 act as negative regulators of the mTOR pathway, and removal leads to hyperactivation of mTOR. mTOR complex 1 (mTORC1) signaling requires activation of the adaptor protein raptor, and mTOR complex 2 (mTORC2), which is largely insensitive to acute rapamycin treatment, requires activation of the rictor protein. Although regulation of mTORC2 is less clearly defined, it has been linked to cytoskeleton organization.

Mammalian target of rapamycin activation is controlled by the PI3K/Akt signaling pathway, where PTEN and the combined TSC1 and TSC2 complex act as negative regulators (**Figure 1**). PI3K is responsible for the production of phosphatidylinositol (3,4,5)-triphosphate (PIP_3_) through the phosphorylation of the biphosphate PIP_2_. PTEN acts in opposition to PI3K, dephosphorylating PIP_3_, resulting in the production of the PIP_2_. PIP_3_ binds PDK-1, allowing PDK-1 to activate Akt; thus, PTEN prevents Akt activation through the depletion of available PIP_3_. When Akt is activated it binds to TSC1/TSC2 complex. Normally when TSC1 and TSC2 are dimerized, the complex binds to RAS homolog enriched in brain (RHEB); however, activated Akt phosphorylates TSC2 and prevents RHEB from binding. In this case, GTP-bound RHEB activates mTOR. Therefore, loss-of-function mutations or removal of PTEN, which leads to hyperactivation of Akt, or loss of TSC1/TSC2, which leads to increased unbound RHEB, can cause an upregulation of mTOR signaling. Furthermore, the mTOR pathway contains two “arms” in which signaling is mediated either by mTOR complex 1 (mTORC1) or mTOR complex 2 (mTORC2). mTORC1 signaling requires activation of the adaptor protein raptor, while mTORC2 signaling requires the rictor adaptor protein. Studies implicate mTORC1 in regulating cell growth and proliferation(for review, see Fingar and Blenis, 2004; Magri et al., 2011), while mTORC2 is implicated in regulating the actin cytoskeleton, soma size, dendritic growth, and dendritic tiling (Jacinto et al., 2004; Koike-Kumagai et al., 2009; Urbanska et al., 2012; Thomanetz et al., 2013).

## INVOLVEMENT OF mTOR IN EPILEPTOGENESIS

### EPILEPSY IS CHARACTERIZED BY RESTRUCTURING OF NEURONAL CIRCUITS IN THE BRAIN

It has been known since the 1980s that epilepsy can be associated with dramatic restructuring of neuronal pathways. Some of the best evidence of this comes from studies of the hippocampus. In tissue surgically resected from patients with temporal lobe epilepsy, hippocampal dentate granule cell mossy fiber axons were found to form *de novo* projections into the dentate inner molecular layer (IML) (Sutula et al., 1989). Subsequent studies revealed that these sprouted axons form excitatory synaptic connections with granule cell dendrites projecting through the IML, creating recurrent loops which have been hypothesized to promote hyperexcitability (for review, see Nadler, 2003). More recently, studies using models of temporal lobe epilepsy have identified ectopic granule cells (Parent et al., 1997; Scharfman et al., 2000; Overstreet-Wadiche et al., 2006), hypertrophied granule cells (Suzuki et al., 1995; Murphy et al., 2011, 2012), granule cells with basal dendrites (Ribak et al., 2000, 2012; Overstreet-Wadiche et al., 2006; Murphy and Danzer, 2011), and granule cells with altered synaptic structure (Pierce and Milner, 2001; Danzer et al., 2010; McAuliffe et al., 2011; Upreti et al., 2012) as common pathologies of the disorder. Interestingly, the dentate gyrus is one of only two regions exhibiting persistent neurogenesis in adulthood, and a majority of the granule cells exhibiting these pathological abnormalities appear to be newborn (Parent et al., 2006; Walter et al., 2007; Kron et al., 2010; Santos et al., 2011). Moreover, these post-seizure born dentate granule cells exhibit accelerated maturity and functional integration into the network; receiving perforant-path input sooner than in a normal brain (Overstreet-Wadiche et al., 2006). While the continued generation of neurons in the dentate may make the region particularly vulnerable to epileptogenic rewiring (Jessberger et al., 2007; Danzer, 2008), restructuring of neuronal circuits is also evident in other brain regions. Rewiring is evident in cortical epilepsy models (for example, Graber and Prince, 2004), and histological and functional mapping studies in humans with a range of epilepsy syndromes support the conclusion that restructuring is a recurring feature of the disease (Bonilha et al., 2012; Fang et al., 2013; Woodward et al., 2013; Xu et al., 2013; Haneef et al., 2014). Changes in neuronal circuitry in epilepsy can be widespread and profound, ranging from alterations in synaptic protein expression and synaptic strength at the subcellular level, to neuronal loss and addition at the cellular level, to altered connectivity between brain regions at the systems level. While such changes are likely to involve many signaling pathways, the mTOR pathway stands out for its ability to regulate cell proliferation, cell survival, cell growth, and synaptic strength. These features make it an appealing candidate for involvement in epileptogenesis.

### HYPERACTIVATION OF THE mTOR SIGNALING PATHWAY CONSISTENTLY PRODUCES EPILEPSY IN ANIMAL MODELS

**FIGURE 2:**
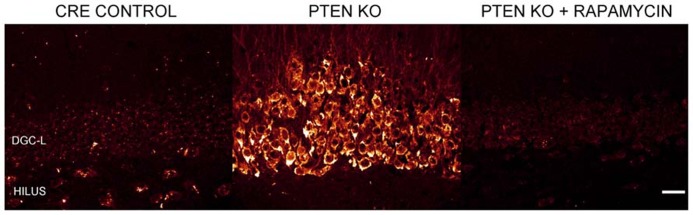
**Dentate granule cells lacking PTEN have hyperactive mTOR signaling.** Confocal maximum projections of the dentate gyrus from control mice, *Gli1-CreER*^T2^ × PTEN^fl/fl^ (PTEN KO) mice, and rapamycin treated PTEN KO mice immunostained for phosphorylated ribosomal protein S6 (pS6; red). pS6, the downstream target of mTOR complex 1, is greatly increased in the dentate granule cell layer (DGC-L) in the PTEN KO animal, demonstrating the upregulation of mTOR signaling with the removal of PTEN. Rapamycin treatment, and thus inhibition of mTOR, prevents the increase in pS6 in PTEN KO mice. Scale bar = 25 μm. Images courtesy of Isaiah Rolle.

Numerous laboratories have examined the impact of PTEN, TSC1, or TSC2 deletions in mouse models. Initial studies examining germline deletions of PTEN (Suzuki et al., 1998; Podsypanina et al., 1999), TSC1 (Kwiatkowski et al., 2002), or TSC2 demonstrated that loss of gene function produced early mortality (Kobayashi et al., 1999; Onda et al., 1999). CNS deletion using conditional approaches also reduced survival in mice (Groszer et al., 2001; Zhou et al., 2009). Therefore, in order to generate viable animals that could be used to study cellular consequences of mTOR hyperactivation, smaller neuronal populations have been targeted with more specific Cre-loxP promoters (Kwon et al., 2001; Marino et al., 2002; Uhlmann et al., 2002; Fraser et al., 2004; Yue et al., 2005; Erbayat-Altay et al., 2007; Meikle et al., 2007; Wang et al., 2007; Zeng et al., 2008, 2011; Way et al., 2012), developmentally timed promoters (Kwon et al., 2006; Zhou et al., 2009), tamoxifen-inducible promoters in which gene removal can be temporally controlled by the researcher (Amiri et al., 2012; Pun et al., 2012), or a combination of these techniques (Abs et al., 2013). As illustrated by **Figure 2**, PTEN removal from dentate granule cells leads to hyperactivation of mTOR, evident as an increase in phosphorylated ribosomal protein S6 (pS6). Rapamycin treatment to prevent mTOR activation blocks the increase in pS6. Although many Cre promoters have been utilized to create the different mouse models, one of the commonalities between these transgenic mice is the incidence of seizures. For example, mice with PTEN removed from 30–60% of mature CA3 neurons, dentate granule cells, and layer III–V cortical neurons developed spontaneous seizures (Kwon et al., 2006), and 40% of mice with PTEN deleted from subgranular and subventricular zone progenitors developed spontaneous seizures (Amiri et al., 2012). Work by Pun et al. (2012) produced the most selective deletion to date, with PTEN removal from as little as 9% of the dentate granule cell population, producing a profound epilepsy syndrome in almost all animals. While PTEN is further upstream from mTOR than TSC1 and TSC2, deletion of either of these regulatory proteins produces similar consequences; although the time course of epileptogenesis varies between models. Removal of TSC1 using *Synapsin-Cre* mice produced knockout (KO) cells throughout the cortex, subcortical gray matter, and hippocampal CA3 and hilar neurons. These animals developed spontaneous seizures by post-natal day 5 and died between 3 and 5 weeks of age (Meikle et al., 2007; Choi et al., 2008). Removal of TSC1 from cortical and subventricular zone neurons in *Emx1-Cre* mice was associated with cortical seizures at post-natal day 13 (Magri et al., 2011; Carson et al., 2012). Similarly, *GFAP-Cre* directed TSC1 or TSC2 deletion from astrocytes and some neurons (Bajenaru et al., 2002; Su et al., 2004) produced seizures between 3 weeks and 2 months of age (Uhlmann et al., 2002; Erbayat-Altay et al., 2007; Zeng et al., 2008, 2010, 2011), while mice with TSC1 or TSC2 removal from radial glial cells commonly had seizures before their death at 3–4 weeks of age (Way et al., 2009; Magri et al., 2013). Finally, recent work by Abs et al. (2013) produced the intriguing result that global deletion of TSC1 from adult animals could result in the onset of spontaneous seizures within 2–9 days. Such rapid epileptogenesis would favor changes that could occur quickly, such as altered neuron physiology. Some “leaky” expression from their Cag-CreERT driver line, however, lead to premature TSC1 deletion from a small number of cells, leaving open the possibility longer term changes among these TSC1 KO cells may accelerate epileptogenesis in the animals. Taken together, these data suggest that mTOR hyperactivation among a wide range of cell types can drive epileptogenesis.

### ENHANCED mTOR SIGNALING IN ACQUIRED EPILEPSY MODELS

Mammalian target of rapamycin pathway upregulation has been described in several animal models of acquired epilepsy. In acquired epilepsy models, epileptogenesis is induced by experimentally injuring the brain using, for example, convulsant drugs or hypoxia. The mTOR pathway is not genetically manipulated in these models, as is the case for TSC and PTEN mutants, so changes in mTOR signaling reflect physiological responses to the injury. It is notable, therefore, that hyperactivation of mTOR signaling is present in acute seizure (Zhang and Wong, 2012), status epilepticus (Okamoto et al., 2010; Macias et al., 2013), traumatic brain injury (Chen et al., 2007), hypoxia (Talos et al., 2012; Sun et al., 2013), and *in vitro* (Berdichevsky et al., 2013) models of epilepsy. Following traumatic brain injury, phosphorylated mTOR was significantly increased after 30 min and remained elevated for 4–24 h (Chen et al., 2007). In the kainic acid status epilepticus model of epilepsy, mTOR signaling increased 1 h after injury, returned to baseline at 24 h, and increased again beginning at 3 days. This second wave lasted for several weeks (Zeng et al., 2009). Persistent upregulation of the pathway is hypothesized to initiate changes in neuronal structure and physiology that contribute to epileptogenesis. Consistent with this idea, administration of the mTOR antagonist rapamycin has been found to inhibit mossy fiber sprouting and mitigate the development of spontaneous seizures in several models of epilepsy (for review, see Wong, 2013a). On the other hand, work by Buckmaster and colleagues found that rapamycin was ineffective at reducing spontaneous seizure frequency in mice (Buckmaster and Lew, 2011; Heng et al., 2013); although mossy fiber sprouting was still blocked (Buckmaster et al., 2009). Whether the discrepant results reflect species differences (mouse vs. rat), model differences, or other factors is not known. Nonetheless, the subset of positive animal findings, combined with promising clinical data in children with TSC (Krueger and Franz, 2008; Muncy et al., 2009; Wong, 2010; Kotulska et al., 2013; Krueger et al., 2013), provide a strong rationale for further investigations.

## REGULATION OF NEURONAL FORM AND FUNCTION BY mTOR

Given the extensive literature demonstrating neuronal rewiring in epilepsy, the ability of mTOR hyperactivation in the CNS to cause epilepsy, the evidence that the mTOR pathway is activated during epileptogenesis, and the role played by mTOR signaling in regulating neuronal plasticity, we decided to explore the consequences of enhanced mTOR signaling on neuronal form and function in the second part of this review. While the exact mechanisms by which excess mTOR signaling promotes epilepsy remain uncertain, we believe that understanding the cellular impacts of this signaling provides novel insights.

### CELL PROLIFERATION

The mTOR signaling pathway is an important regulator of developmental and adult neurogenesis. Cell proliferation is impaired in mTOR KO mice, as well as RAPTOR and RICTOR KO mice, in which mTORC1 or mTORC2 signaling, respectively, is blocked. Loss of either mTOR, raptor, or rictor results in embryonic lethality, possibly in part due to impaired cell proliferation (Gangloff et al., 2004; Murakami et al., 2004; Guertin et al., 2006; Shiota et al., 2006; Cloetta et al., 2013). Conversely, embryonic deletion of PTEN from CNS stem/progenitor cells decreases apoptosis and increases neuron numbers compared to controls (Groszer et al., 2001). TSC deletions also promote cell proliferation, with the hallmark feature of the human disease being cortical tubers. The tubers are accumulations of non-malignant cells that exhibit abnormal proliferation, differentiation, and migration. In developing animals, TSC1 deletion from radial glial cells disrupts the subventricular proliferative zones and leads to megalencephaly (Magri et al., 2013). In the adult brain, effects of mTOR dysregulation on neuronal proliferation are less pronounced since adult neurogenesis is limited to olfactory and hippocampal granule neurons, but it is clear that mTOR plays an important role in regulating the generation of these cells (Hartman et al., 2013; Magri and Galli, 2013). Conditional deletion of PTEN from the subventricular zone progenitors, for example, increased proliferation of neurons populating the olfactory bulb (Gregorian et al., 2009), while inhibiting the PI3K/PTEN–mTOR pathway (using rapamycin and the PI3K inhibitor LY294002) led to reduced subventricular zone proliferation (Peltier et al., 2007; Paliouras et al., 2012). Rapamycin had a similar effect on subgranular zone production of granule cells (Raman et al., 2013). These data suggest that proliferation of adult-generated dentate granule cells would be increased with mTOR hyperactivation due to PTEN, TSC1, or TSC2 deletion. Consistent with this, investigators examining PTEN KO mice, including the *GFAP-Cre* and *Nestin-CreER*^T2^ lines, have reported increased cell proliferation *in vivo* at early time points (up to 4 months; Fraser et al., 2004; Amiri et al., 2012). However, increased production of granule cells may be temporary, as proliferation rates declined at later time points (Amiri et al., 2012). It is possible that the accelerated differentiation, which contributes to increased proliferation at early time points, depletes the subgranular zone stem cell pool at later time points. Ventricular zone progenitors, on the other hand, do not appear to exhibit the same limitation (Gregorian et al., 2009).

Increased neurogenesis from enhanced mTOR signaling can have profound effects when the altered signaling occurs early in development and is widespread, with the excess neurons contributing to gross brain abnormalities like hemimegalencephaly. Altered neurogenesis may also contribute to more subtle defects, like heterotopias and dysplasias. These small clusters of abnormal neurons often develop into epileptic foci, as evidenced by electroencephalography (EEG) studies and the abolition of seizures in many patients following surgical removal of the abnormal cells (for review, see Hauptman and Mathern, 2012). Interestingly, evidence has recently emerged suggesting that prenatal infection with human papillomavirus (HPV) may disrupt mTOR signaling, leading to the development of focal cortical dysplasia type II. The HPV oncoprotein E6 was found in resected human dysplasias, but not surrounding tissue, and transfection of fetal mouse brain with an E6 expression vector potently activated mTOR targets (Chen et al., 2012; Lim and Crino, 2013; Wong, 2013b; Yasin et al., 2013). Additional subtle effects of increased mTOR signaling in acquired epilepsy may include the burst of neurogenesis observed following epileptogenic brain injury in rodents (Parent et al., 1997). mTOR signaling promotes neurogenesis, and large numbers of these newborn cells integrate abnormally, raising the possibility that these new neurons may by epileptogenic (Scharfman and Hen, 2007; Scharfman and McCloskey, 2009; Parent and Kron, 2012). If true, it is possible that reduced seizure frequency following mTOR inhibition (for review, see Ryther and Wong, 2012) may be partly mediated by reduced neurogenesis. Finally, evidence that subgranular zone progenitors can be depleted following prolonged mTOR hyperactivation raises the possibility that epileptogenesis and seizures could accelerate the decline of this important cell population (Amiri et al., 2012). In some rodent epilepsy models, granule cell neurogenesis is increased in the first few weeks and months after the insult, but persistent decreases in neurogenesis have been observed when the animals are examined months later (Hattiangady and Shetty, 2010; Danzer, 2012).

### SOMATIC HYPERTROPHY

**FIGURE 3:**
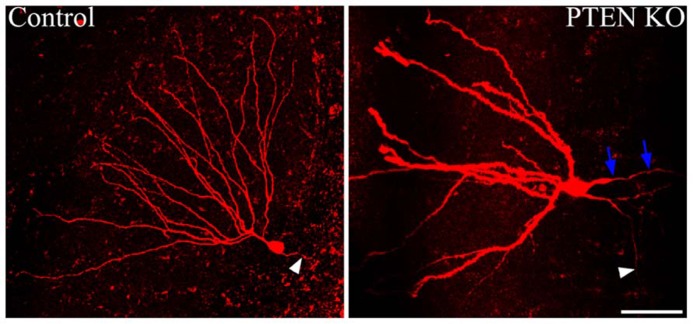
**Dentate granule cells lacking PTEN exhibit structural abnormalities.** Confocal maximum projections of control and *Gli1-*CreER^T2^ × PTEN^fl/fl^ (PTEN KO) granule cells, filled with biotin and labeled with Alexa-Fluor 594-conjugated streptavidin. The PTEN KO cell exhibits somatic hypertrophy and dramatically increased dendritic thickness compared to the control dentate granule cell. Additionally, basal dendrites are commonly found on the PTEN-negative cells, here indicated by blue arrows. White arrowheads highlight the mossy fiber axon extending toward the hilus from each of the two cells. Scale bar = 50 μm. Images courtesy of Victor R. Santos and Dr. Raymund Y. K. Pun.

Somatic hypertrophy among neurons has been one of the most consistent findings in cells with increased mTOR signaling (**Figure 3**). In animal models, somatic hypertrophy has been observed following deletion of TSC1 (Meikle et al., 2007; Zeng et al., 2008; Zhou et al., 2009; Tsai et al., 2012), TSC2 (Zeng et al., 2011; Tsai et al., 2014), and PTEN (Backman et al., 2001; Groszer et al., 2001; Kwon et al., 2001, 2003, 2006; Fraser et al., 2004; Ljungberg et al., 2009; Amiri et al., 2012; Pun et al., 2012). Hypertrophy has been detected in hippocampal granule cells (Backman et al., 2001; Kwon et al., 2001, 2003, 2006; Fraser et al., 2004; Amiri et al., 2012; Pun et al., 2012), cortical neurons (Fraser et al., 2004; Kwon et al., 2006; Meikle et al., 2007; Ljungberg et al., 2009; Zhou et al., 2009), and purkinje cells (Tsai et al., 2012; Thomanetz et al., 2013). Treatment with rapamycin or CCI-779 to inactivate mTOR prevents the somatic hypertrophy (Kwon et al., 2003; Zeng et al., 2008, 2011; Zhou et al., 2009), and similarly, inhibition of mTORC2 signaling by deletion of RICTOR reduces neuronal size (Thomanetz et al., 2013). Somatic enlargement associated with mTOR hyperactivation is seen in human TORopathies, and is particularly prominent in patients with TSC, who exhibit giant cells within the tubers that characterize the disease (White et al., 2001). Enlarged neurons are also present in focal cortical dysplasia, in which cytomegalic neurons and “balloon cells” are characteristic. Altogether, these data support the conclusion that mTOR dysregulation promotes abnormal cell growth.

Increased soma size can alter neuronal function at both microscopic and macroscopic levels. At the single cell level, increased soma area would be predicted to decrease cellular input resistance and increase total cell capacitance. These predictions were upheld by Luikart et al. (2011) in work examining hippocampal granule cells following small hairpin RNA (shRNA)-mediated PTEN knockdown. Knockdown cells exhibited neuronal hypertrophy, decreased input resistance, and increased capacitance. Similar findings were reported for TSC1 KO cells (Bateup et al., 2011, 2013b). At the macroscopic level, increased soma size in PTEN, TSC1, or TSC2 KO mice contributes to gross distortion of brain structure. Specifically, cellular hypertrophy (potentially combined with increased neurogenesis) leads to progressive macrocephaly with increased brain size in mouse models, usually accompanied by disorganization of cell layers (Groszer et al., 2001; Kwon et al., 2001, 2003, 2006; Fraser et al., 2004; Zeng et al., 2008, 2011; Zhou et al., 2009; Amiri et al., 2012). The increase in cell size and cell layer disorganization can cause compression of other brain areas, such as hippocampal CA1 in the *NSE-Cre* PTEN KO mouse (Kwon et al., 2006). These dramatic disruptions likely contribute to functional deficits in the animal models and humans with related conditions like hemimegalencephaly.

### CELL POLARIZATION

Establishing proper polarity is critical step in neuronal differentiation, and mTOR signaling appears to play a modulatory role in this process. In the rodent hippocampal dentate gyrus, granule cells typically possess only apical dendrites, which extend from the soma into the dentate molecular layer. When the mTOR pathway is hyperactivated in granule cells by PTEN deletion, however, large numbers of granule cells develop aberrant basal dendrites (**Figure 3**; Kwon et al., 2006; Pun et al., 2012). Basal dendrites are also a feature of rodent epilepsy models, where the abnormal processes project into the dentate hilus. These hilar-projecting dendrites are innervated by the mossy fiber axons of neighboring granule cells, creating recurrent excitatory loops which may contribute to hyperexcitability (Ribak et al., 2000, 2012; Thind et al., 2008). Granule cell basal dendrites that develop after PTEN deletion have spines apposed to presynaptic terminals of mossy fiber axons, suggesting they also mediate the formation of recurrent circuits (Pun et al., 2012). These recurrent connections are thought to contribute to hyperexcitability and seizure generation in PTEN KO animals.

Whether PTEN deletion disrupts cell polarization directly or indirectly is not clear. Immature granule cells frequently exhibit transient basal dendrites, which are eliminated as the cells mature (for review, see Rahimi and Claiborne, 2007). Retraction of these basal dendrites appears to be regulated by activity, since increasing neuronal activity *in vitro* can lead to their persistence (Nakahara et al., 2009). It is possible, therefore, that basal dendrites in PTEN KO mice are a consequence of increased neuronal activity in these animals (or the seizures that ultimately develop), although additional studies will be needed to test the idea.

### AXON GROWTH

**FIGURE 4:**
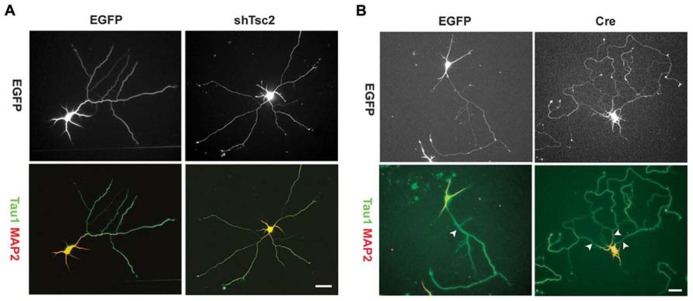
**Tuberous sclerosis complex deficiency induces multiple axons in culture.** Knockdown of Tsc2 by shRNA (shTsc2) or knockout of Tsc1 (Cre) induced multiple axons in vitro. **(A)** E18 rat hippocampal neurons transfected with either enhanced green fluorescent protein (EGFP) alone or EGFP together with Tsc2 shRNA. Tsc2 knockdown induced multiple axons, all of which are positive for Tau1. **(B)** E17 mouse hippocampal neurons from Tsc1^lox/flox^ embryos transfected with EGFP alone or EGFP together with Cre. Arrowheads indicate axons positive for Tau1. Scale bars = 20 μm. Reprinted with permission from Choi et al. (2008).

**FIGURE 5:**
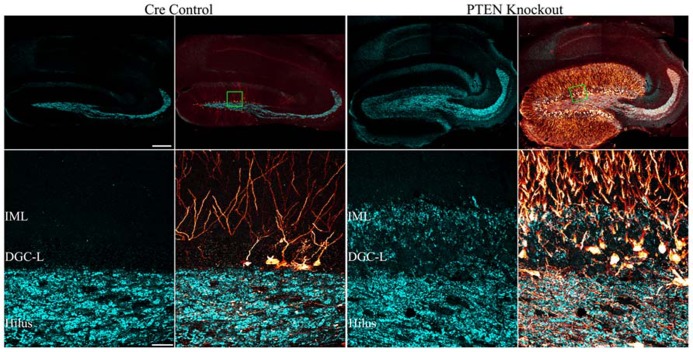
**Mossy fiber sprouting is present in mice with PTEN removal from dentate granule cells.** Confocal maximum projections of hippocampi from tamoxifen-treated control and *Gli1-CreER*^T2^ × PTEN^fl/fl^ (PTEN KO) mice immunostained for GFP (red) and zinc-transporter 3 ZnT3 (cyan) are shown. ZnT3-labeling reveals the normal dentate granule cell mossy fiber axon terminal field (hilus and stratum lucidum) in the control animal, while mossy fiber sprouting into the dentate granule cell layer (DGC-L) and inner molecular layer (IML) is evident in the knockout animal (green boxes in the top row correspond to high resolution images shown in the bottom row). Scale bars = 200 μm (top row) and 30 μm (bottom row). Reprinted with permission from Pun et al. (2012).

Axon growth appears to be promoted by the mTOR pathway, and disrupted mTOR activation can lead to irregular axon development. Overexpression of TSC1 or 2 *in vitro* – thus decreasing mTOR signaling – results in fewer neurons possessing an axon. Conversely, KO of TSC1 or knockdown of TSC2 increases the number of neurons with multiple axons (**Figure 4**), and rapamycin treatment was found to mitigate this effect in the TSC2 knockdown cells (Choi et al., 2008). Consistent with this *in vitro* work, deletion of TSC1 in a *Synapsin-Cre* TSC1 KO mouse model led to ectopic axon growth in cortex (Choi et al., 2008). PTEN removal *in vivo* also increased axon growth. PTEN deletion from dentate granule cells, occurring during development or after the cells mature, led to progressive enlargement of the granule cell mossy fiber axon tract (Kwon et al., 2006; Amiri et al., 2012). Moreover, *NSE-Cre* TSC1 KO mice exhibited increased thickness of the mossy fiber tract, while rapamycin administration reduced tract thickness (Zhou et al., 2009). Finally, work by Pun et al. (2012) demonstrated that selective deletion of PTEN from granule cells provoked aberrant axon sprouting into the dentate IML (mossy fiber sprouting, **Figure 5**). Together, these data suggest that mTOR signaling is involved in axon outgrowth, and disruption of this pathway can lead to axon path thickening (presumably due to larger numbers of axons/axon collaterals).

The mechanism by which mTOR signaling promotes the sprouting of granule cell mossy fiber axons into the dentate IML remains unclear. In acquired epilepsy models, rapamycin has been consistently found to block mossy fiber sprouting, suggesting that increased mTOR signaling may mediate this pathology directly (Zeng et al., 2009; Huang et al., 2010; Buckmaster and Wen, 2011). However, deletion of PTEN from dentate granule cells does not always produce mossy fiber sprouting. In fact, mossy fiber sprouting was only detected in *Gli-CreER*^T2^ PTEN KO mice when the number of granule cells without PTEN exceeded ≈15% of the entire granule cell population (Pun et al., 2012). Interestingly, some animals with fewer PTEN KO cells developed seizures, but not mossy fiber sprouting: evidenced both by a lack of mossy fiber terminal immunolabeling in the IML, and an absence of green fluorescent protein (GFP)-labeled PTEN KO cells axons in this same region (Pun et al., 2012). These latter findings suggest that mossy fiber sprouting is an emergent property of mTOR hyperactivation. Restated, it would appear than mTOR hyperactivation in individual granule cells is not sufficient to drive mossy fiber sprouting, but large numbers of PTEN KO granule cells acting together do produce mossy fiber sprouting, suggesting that secondary processes are involved. It has been proposed that mossy fiber sprouting is triggered by mossy cell loss, as the degree of sprouting is positively correlated with the extent of mossy cell loss (Jiao and Nadler, 2007). Mossy cell loss in the absence of seizures, however, does not cause mossy fiber sprouting (Jinde et al., 2012), suggesting that while the two phenomena are correlated, they may not be causally linked. The ability of rapamycin to block mossy fiber sprouting in acquired epilepsy models, and the apparent inability of increased mTOR signaling to drive mossy fiber sprouting unless a threshold level of KO cells is met, raises interesting questions about when and where rapamycin is acting.

The net effect of increased axon sprouting following increased mTOR activation is uncertain (Buckmaster, 2012). Although granule cell mossy fiber sprouting is not required to produce epilepsy in mTOR hyperactivation models, increased sprouting of granule cells may contribute to epilepsy progression in the animals by promoting the formation of recurrent excitatory circuits. Alternatively, mossy fiber sprouting may play a compensatory role by promoting feedback inhibition. In addition to contacting excitatory granule cells, sprouted mossy fibers have also been observed to innervate inhibitory interneurons in animal models of epilepsy (Frotscher et al., 2006; Sloviter et al., 2006). Enhanced mTOR signaling may also increase inhibition by promoting sprouting of other neuronal types. Work by Buckmaster and Wen (2011) demonstrated that inhibiting mTOR with rapamycin in the pilocarpine model of epilepsy can reduce axon sprouting of somatostatin-expressing GABAergic interneurons, raising the possibility that mTOR-mediated sprouting in epilepsy may exert both pro-excitatory and homeostatic effects, depending on the cell type affected.

### DENDRITIC GROWTH

Signaling through the PI3K/PTEN–mTOR pathway promotes dendritic growth and branching. In cultured hippocampal neurons, constitutively activating PI3K, or RNA interference (RNAi) knockdown of PTEN, resulted in increased dendrite number, branching complexity, and total dendrite length (Jaworski et al., 2005). Moreover, the effects appear to be mediated by mTOR, as rapamycin administration blocked abnormal dendritic growth following constitutive PI3K activation and PTEN knockdown (Jaworski et al., 2005). *In vitro*, the effects of PI3K activation on dendritic length and complexity appear to be limited to immature neurons, since mature neurons did not exhibit changes in dendritic structure (Cuesto et al., 2011). Removal of PTEN *in vivo* supports *in vitro* data; PTEN removal from dentate granule cells resulted in dendritic hypertrophy, elongated dendrites, and increased arborization among developing neurons (**Figure 3**; Kwon et al., 2006; Amiri et al., 2012; Pun et al., 2012). Removal of TSC1 from dentate granule cells increased molecular layer thickness, suggesting dendritic hypertrophy akin to the PTEN KO mice (Zhou et al., 2009). Rapamycin can reduce dendritic overgrowth in PTEN KO mice, further specifying mTOR signaling in dendritic size and branching (Zhou et al., 2009). Thus, it appears that mTOR controls growth of the soma and neuronal processes, and continued activation causes processes to thicken and elongate.

The structure of the dendritic tree is thought to be critical for precise signal processing. Therefore, changes in dendritic thickness associated with mTOR hyperactivation have the potential to alter synaptic potential integration. Theoretical models of synaptic potential attenuation use dendrite diameter in the estimation of the current flow. Thicker dendrites facilitate signal spread toward the soma compared to thinner dendrites, although thicker dendrites would also predict smaller excitatory post-synaptic potentials (EPSPs), as the larger processes should have lower input resistance (for review, see Rall, 2011). The increased surface area provided by larger dendrites increases the post-synaptic “real estate” available for innervation, potentially constituting one mechanism by which neurons regulate their synaptic input. Elucidating the net effect of thicker dendrites will require additional studies, and might vary by neuron and condition.

### CELL INTRINSIC EFFECTS, PRIMARY NETWORK EFFECTS, AND SECONDARY NETWORK EFFECTS

**FIGURE 6:**
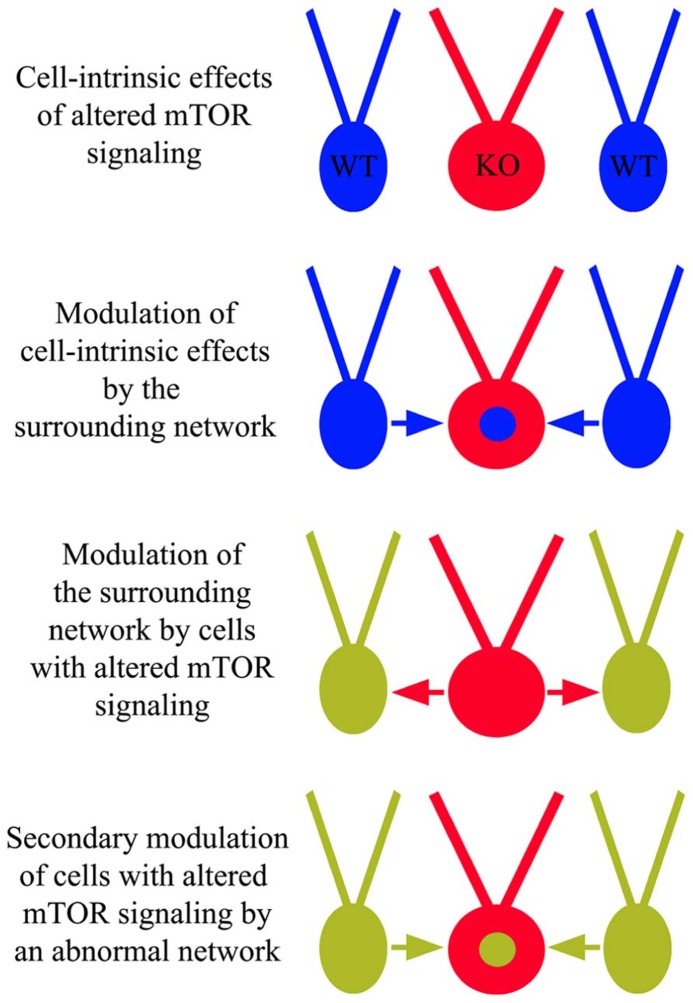
**Cell-intrinsic effects of altered mTOR signaling and network influence.** In addition to cell-intrinsic effects from disrupted mTOR signaling in cells lacking PTEN, TSC1, or TSC2 (red “KO” cells), these cells may respond inappropriately to surrounding wild type (WT) cells and/or cause WT cells to behave abnormally. These altered dynamics may produce an abnormal network, facilitating epileptogenesis.

Anatomical and physiological studies examining the impact of mTOR hyperactivation following PTEN or TSC deletion have produced a number of conflicting findings. Some of these discrepancies likely involve the usual suspects, such as cell and gene-specific effects, but an emerging story suggests much of this complexity may reflect a role for the mTOR pathway in regulating network homeostasis and plasticity. The ability of mTOR to regulate synaptic structure and function clearly places the pathway in a position to impact how neurons respond to activity levels in their environment. This observation has two important implications. Firstly, it suggests that cells with disrupted mTOR signaling may respond inappropriately to input from other neurons in a fashion that is context dependent. Restated, different environments may produce different types of changes in PTEN or TSC KO cells. Secondly, since PTEN and TSC KO cells exhibit altered activity patterns, they can influence the activity of surrounding neurons, and these activity changes in the network may in turn alter the behavior of the KO cells (**Figure 6**). If correct, it may be more useful to view the mTOR signaling pathway as a means by which a neuron regulates its “social” behavior in a network. In the following sections we will present examples that demonstrate typical cell and gene-specific differences that account for variability, and more intriguing evidence of primary and secondary network effects on KO cell behavior.

### CELL TYPE AND GENE-SPECIFIC EFFECTS: LONG-TERM DEPRESSION

In addition to changes in cell structure, mTOR hyperactivation is associated with changes in synaptic plasticity. Physiological changes that have been detected include a decrease in long-term depression (LTD). LTD is defined by a reduction in the efficacy of a synapse and is typically induced experimentally by low frequency stimulation. Two mechanistically distinct types of LTD co-exist at hippocampal synapses: a metabotropic glutamate receptor (mGluR)-dependent form and a *N*-methyl-D-aspartate (NMDA) receptor (NMDAR) dependent form (Mulkey and Malenka, 1992; Bolshakov and Siegelbaum, 1994; Oliet et al., 1997). mGluR-LTD, which requires dendritic protein synthesis, can be selectively induced by (*S*)-3,5-dihydroxyphenylglycine (DHPG), an agonist that activates group I mGluRs (Palmer et al., 1997; Fitzjohn et al., 1999; Schnabel et al., 1999; Huber et al., 2001). Activation of group I mGluRs triggers the PI3K–Akt–mTOR signaling cascade, which increases Akt and mTOR phosphorylation in CA1 dendrites. Moreover, treatment with rapamycin or PI3K inhibitors block DHPG-induced LTD (Hou and Klann, 2004), clearly implicating mTOR signaling in the process. Paradoxically, hyperactivation, like inhibition, of mTOR seems to mostly impair LTD; possibly as a secondary consequence of seizures in the animals (Kirschstein et al., 2007). The targeted cell type also appears to be important. Specifically, Takeuchi et al. (2013) showed mGluR-LTD was impaired at all ages in PTEN KO mice at the perforant path ≫ granule cell synapse, but not CA3 ≫ CA1 synapses. Interestingly, PTEN was deleted from dentate granule cells in these animals, whereas the PTEN gene was left intact in CA1 pyramidal cells, suggesting that mTOR hyperactivation in the post-synaptic cell may be responsible for the impairment in mGluR-LTD. Supporting this conclusion, TSC1 deletion specifically in CA1 neurons did impair mGluR-LTD in these same cells (Bateup et al., 2011). The location of mTOR hyperactivation, therefore, can be an important modulator of effect.

Interestingly, although PTEN is best known for its ability to regulate mTOR signaling, some of its effects on LTD may be mTOR independent. PTEN is required for the expression of NMDAR-LTD (Wang et al., 2006; Jurado et al., 2010), but TSC1 does not appear to be important for this form of LTD. Specifically, Sperow et al. (2012) found NMDAR-LTD to be substantially reduced at the CA3 ≫ CA1 synapse when PTEN deletion was targeted to post-synaptic CA1 neurons, while Bateup et al. (2011) demonstrated that NMDAR-dependent LTD was normal in mice in which TSC1 was deleted from CA1 neurons. Both mouse models exhibit hyperactivation of mTOR. PTEN, however, is much further upstream in the mTOR signaling pathway, with potentially distinct consequences. In fact, Jurado et al. (2010) demonstrated the NMDAR activation triggers an association between PTEN and the synaptic scaffold, anchoring PTEN at the post-synaptic terminal. Furthermore, PTEN removal, and not TSC1 deletion, would affect the PIP_3_ and PI3K influenced pathways. PI3Kγ is critical for NMDAR-LTD, and it is thought to signal via the activation of p38 MAPK and not the mTOR pathway, potentially explaining why NMDAR-LTD is normal in TSC1 KO mice and impaired in PTEN KO mice (Kim et al., 2011). mTOR hyperactivation by TSC1 deletion likely facilitates changes in network dynamics through the loss of mGluR-LTD; however, it appears that PTEN deletion would have more serious consequences for a neuron and its network due to impairments in both mGluR-LTD and NMDAR-LTD. Gene specific effects, therefore, are likely to account for some of the controversy in the mTOR field.

### CELL INTRINSIC vs. SECONDARY EFFECTS: DENDRITIC SPINES

Dendritic spines are the major points of excitatory contact between neurons, and measures of spine density are frequently used as an indirect measure of excitatory input. Notably, inhibitory neuron synapses typically form on dendritic shafts or somata, and therefore are not revealed by light microscopy approaches. Studies examining mTOR regulation of dendritic spines and synapses support a pro-excitatory role for the signaling pathway; although complex mechanisms may be involved. PTEN deletion has been associated with an increase in spine density in most studies (Kwon et al., 2006; Pun et al., 2012), however, Haws et al. (2014) recently observed no change in spine density following PTEN deletion from hippocampal granule cells, and actually observed a decrease in spine density following PTEN knockdown in basolateral amygdala. Despite the reduction in spine density, the changes were associated with an increase in miniature EPSC (mEPSC) frequency and amplitude (Haws et al., 2014). The authors suggest that the discrepancy reflects a shift from thin spines to mushroom spines, the latter being associated with stronger synapses. Similarly, Luikart et al. (2011) found that PTEN knockdown using shRNA injection in mice caused increased mEPSCs and spontaneous post-synaptic currents in dentate granule cells. Deletion of TSC1 and TSC2 has been shown to reduce dendritic spine density among cortical neurons *in vivo* (Tavazoie et al., 2005; Meikle et al., 2008) and hippocampal pyramidal cells in organotypic explant cultures (Tavazoie et al., 2005; Meikle et al., 2008); Since both TSC and PTEN KO mice develop epilepsy, however, many of the changes observed in these animals likely reflect phenomena secondary to the occurrence of increased activity and spontaneous seizures (Wong and Guo, 2013). Indeed, it is well established that seizures alone can induce spine loss (Swann et al., 2000; Brewster et al., 2013; Singh et al., 2013), perhaps accounting for the cortical spine loss observed by Meikle et al. (2008) in TSC1 KO mice. Consistent with this idea, when Bateup et al. (2011) induced sparse TSC1 deletion from CA1 pyramidal cells *in vivo*, which did not induce epilepsy, spine reductions were absent.

### CELL INTRINSIC vs. SECONDARY EFFECTS: BASAL SYNAPTIC TRANSMISSION

At entorhinal cortex ≫ dentate granule cell synapses, basal synaptic transmission has been found to exhibit complex temporal changes in *Nse-Cre* PTEN KO mice, strongly suggesting that secondary changes are having a significant impact on physiology in these animals. The *Nse-Cre* transgene produces robust PTEN deletion from dentate granule cells and CA3 pyramidal cells, with relative sparing of CA1 (Takeuchi et al., 2013). In young *Nse-Cre* PTEN KO mice, perforant path stimulation to evoke EPSCs in the dentate gyrus produced input/output relationships that were indistinguishable from controls. Middle-aged animals, on the other hand, exhibited enhanced input/output relationships (Kwon et al., 2006; Takeuchi et al., 2013). As the animals aged, however, the differences resolved, with responses again becoming indistinguishable from controls. Although the mechanism is uncertain, the return to baseline may reflect compensatory modifications to the network (Takeuchi et al., 2013). The temporal dynamics of the physiological changes parallel morphological changes in these animals, with young animals appearing relatively normal, middle aged animal beginning to show subtle changes in structure such as increased spine density, and older animals exhibiting gross structural changes and seizures.

### CELL INTRINSIC vs. SECONDARY EFFECTS: LONG-TERM POTENTIATION

Long-term potentiation (LTP) is considered to be one of the cellular mechanisms that underlies learning. During LTP, synaptic strength is increased between connected neurons, and is typically induced by high frequency stimulation. mTOR activation appears to be involved in late phase LTP (L-LTP), which is long-lasting and dependent on protein synthesis and gene transcription, but not in early phase LTP, which is short-lasting and requires activation of protein kinase C. Inhibiting mTOR with rapamycin has consistently been shown to decrease L-LTP (Terashima et al., 2000; Tang et al., 2002; Cammalleri et al., 2003; Cracco et al., 2005; Tsokas et al., 2005; Vickers et al., 2005; Stoica et al., 2011) and a recent study of *CamKIIα-Cre* Rictor KO mice found a similar L-LTP deficit, implicating mTORC2 (Huang et al., 2013). Paradoxically, although inhibition of mTOR blocks L-LTP, the majority of studies examining PTEN and TSC KO mice have not found that hyperactivation of mTOR facilitates it. For example, L-LTP was decreased in both *GFAP-Cre* and *CamKIIα-Cre* PTEN KO mice (Fraser et al., 2008; Sperow et al., 2012), and TSC1 KO mice exhibited a similar phenotype (Zeng et al., 2007; Abs et al., 2013). These discordant results, however, may be explained by the occurrence of seizures in the animals. Seizure activity has been shown to impair LTP by shifting the majority of synapses to the “potentiated” state, precluding further enhancement (Beck et al., 2000; Goussakov et al., 2000; Meador, 2007). Consistent with this idea, Zeng et al. (2007) were able to partially reverse the LTP deficit in their TSC1 KO mice by applying the NMDA antagonist APV, suggesting that excessive activation of NMDARs contributes to this impairment. The study from Takeuchi et al. (2013), in which *Nse-Cre* PTEN KO mice were examined at multiple time points, provides further support for the idea that LTP impairment may be a secondary effect of mTOR hyperactivation. Similar to the prior studies, in middle-aged animals, PI3K-dependent LTP was impaired at perforant path ≫ dentate granule cells synapses and Schaffer collateral ≫ CA1 synapses (Takeuchi et al., 2013). In contrast to the prior studies, however, when young animals were examined, they were found to have elevated LTP at perforant path ≫ dentate granule cell synapses. A pre-seizure increase in neuronal excitability of CA1 pyramidal cells and a reduced threshold for L-LTP in the Schaffer collateral pathway has also been noted in TSC1 KO mice following an acute deletion (Abs et al., 2013). Enhanced LTP, therefore, may be a primary effect of increased mTOR activation, while LTP impairments may reflect the consequences of secondary phenomena, such as seizures or structural abnormalities that alter network properties and KO cell behavior.

### CELL INTRINSIC vs. PRIMARY NETWORK EFFECTS

Approaches in which PTEN or TSC deletion is sparsely induced in neuronal populations have significant advantages over more widespread deletions, as secondary changes, such as seizures, can be avoided. A “sparse deletion” approach was used by Sabatini’s group in an elegant series of papers in which TSC1 was deleted from brain and tissue of TSC1^flox/flox^ mice using a Cre-expressing virus. In the first study, TSC1 was deleted from pyramidal cells in organotypic hippocampal explant cultures (Tavazoie et al., 2005). TSC1 KO cells in this tissue exhibited increased spine length and spine head width, decreased spine density and increased mEPSC amplitude with no change in frequency. In the follow-up study, a similar approach was used *in vivo*, but with very different results (Bateup et al., 2011). Pyramidal cells in this study did not exhibit any changes in spine morphology or density, mEPSC amplitude was unchanged, and mEPSC frequency was increased. Interestingly, mGluR-LTD was impaired, removing an important mechanism for mitigating excess excitation from TSC1 KO cells. Organotypic hippocampal cultures exhibit much higher activity levels than hippocampus *in vivo*, and develop seizure-like activity over time (Dyhrfjeld-Johnsen et al., 2010). TSC1 KO cells *in vitro*, therefore, experience a very different environment than similar KO cells *in vivo*, potentially explaining the contrasting behavior of the two populations. Consistent with this idea, Bateup et al. (2013a) were able to demonstrate in dissociated cultures of TSC1 KO neurons that pharmacologically blocking activity prevented many of the gene expression changes in these cells, suggesting that they occur secondarily to the increased activity levels evident in the TSC1 KO cultures.

While the majority of studies examining mTOR effects on neuronal function have examined excitatory signaling, it is important not to downplay the potential significance changes in inhibitory signaling might have. As a case in point, Bateup et al. (2013b) recently demonstrated that although isolated TSC1 KO CA1 pyramidal cells exhibit enhanced mEPSC frequency and impaired mGluR-LTD *in vivo*, the KO cells were intrinsically *less excitable*, leading to little net change in excitatory synaptic drive. Rather, they found that network hyperexcitability was likely produced by a significant reduction in inhibitory drive. TSC1 KO cells in these animals exhibited decreased frequency and amplitude of miniature inhibitory post-synaptic currents (mIPSCs) and reduced amplitude of evoked IPSCs. These effects could be blocked with rapamycin treatment, confirming that they are mediated by mTOR (Bateup et al., 2013b). Altered excitatory/inhibitory balance in the brain, in this case favoring increased excitability via reduced inhibition, might be an important mechanism by which changes in mTOR signaling promote epileptogenesis.

## SUMMARY

Hyperactivation of the mTOR pathway produces a constellation of changes predicted to promote epileptogenesis and hyperexcitability. mTOR signaling can promote adult neurogenesis in the hippocampus, and many of these new cells can integrate abnormally. Increased neurogenesis occurs in most animal models of temporal lobe epilepsy and is suspected of contributing to epileptogenesis (Jung et al., 2004, 2006; Hester and Danzer, 2013). In transgenic mice with inactivation or removal of PTEN, TSC1, or TSC2, cell populations with the resulting mTOR hyperactivation have many common structural abnormalities, including somatic and dendritic hypertrophy, aberrant basal dendrites, and enlargement of axon tracts. Morphological changes are consistent with increased synaptogenesis and recurrent circuit formation – both changes predicted to be epileptogenic. At the synaptic level, transgenic mouse models with mTOR hyperactivation frequently, but not always, exhibit enhanced synaptic transmission and plasticity. The mTOR signaling pathway plays a critical role in regulating how a neuron responds to the network in which it is embedded, and correspondingly, the impact of disrupting the pathway appears to exhibit network specific effects. Moreover, large populations of PTEN and TSC KO cells have the ability to induce secondary changes among the networks they influence, which in turn may produce tertiary, feedback changes among the KO cells. The complexity of this system creates significant hurdles for understanding the mTOR pathway, and is likely responsible for many of the paradoxical findings, but future studies are likely to provide important new insights into the acute and chronic effects of disrupted mTOR signaling in the many diseases in which the path is now implicated.

## Conflict of Interest Statement

The authors declare that the research was conducted in the absence of any commercial or financial relationships that could be construed as a potential conflict of interest.
